# The Use of Dual Cyclodextrin Chiral Selector Systems in the Enantioseparation of Pharmaceuticals by Capillary Electrophoresis: An Overview

**DOI:** 10.3390/molecules26082261

**Published:** 2021-04-14

**Authors:** Gabriel Hancu, Lajos Attila Papp, Gergő Tóth, Hajnal Kelemen

**Affiliations:** 1 Department of Pharmaceutical and Therapeutic Chemistry, Faculty of Pharmacy, University of Medicine, Pharmacy, Science and Technology “George Emil Palade” of Târgu Mureș, 540142 Târgu Mureș, Romania; gabriel.hancu@umfst.ro (G.H.); hajnal.kelemen@umfst.ro (H.K.); 2 Department of Pharmaceutical Chemistry, Semmelweis University, H-1092 Budapest, Hungary; toth.gergo@pharma.semmelweis-univ.hu

**Keywords:** capillary electrophoresis, chiral separation, chiral selectors, cyclodextrins, dual cyclodextrin system

## Abstract

Cyclodextrin (CD) derivatives are the most efficient and frequently used chiral selectors (CSs) in capillary electrophoresis (CE). There are situations when the use of a single CD as CS is not enough to obtain efficient chiral discrimination of the enantiomers; in these cases, sometimes this problem can be resolved using a dual CD system. The use of dual CD systems can often dramatically enhance enantioseparation selectivity and can be applied for the separation of many analytes of pharmaceutical interest for which enantioseparation by CE with another CS systems can be problematic. Usually in a dual CD system an anionic CD is used together with a neutral one, but there are situations when the use of a cationic CD with a neutral one or the use of two neutral CDs or even two ionized CDs can be an efficient solution. In the current review we present general aspects of the use of dual CD systems in the analysis of pharmaceutical substances. Several examples of applications of the use of dual CD systems in the analysis of pharmaceuticals are selected and discussed. Theoretical aspects regarding the separation of enantiomers through simultaneous interaction with the two CSs are also explained. Finally, advantages, disadvantages, potential and new direction in this chiral analysis field are highlighted.

## 1. Introduction

More than half of the pharmaceutical substances currently used in therapy are chiral, however only about 25% are used in the form of a pure enantiomer. It is known that usually the desired pharmacological effect is restricted to only one of the enantiomers, called eutomer, while the other, called distomer, can be less potent, exhibit different pharmacological activity and sometimes can even be responsible for the adverse effects reported after racemic mixture administration [1].

In the past 25 years the number of pure enantiomers introduced in therapy increased constantly, after the publication in 1992 of FDA regulations regarding the development of new stereoisomeric drugs. Currently the properties of individual enantiomers of a racemic mixture should be studied and characterized individually; and the stereoisomeric composition of an optically active drug must be verified from pharmacokinetic, pharmacologic, and toxicologic point of view [2].

These regulations have major implications in the development of new analytical methods for chiral drug control. The development of efficient and reliable chiral separation techniques is a necessity in assessing enantioselective synthesis, racemization processes, verifying enantiomeric purity and in pharmacokinetic studies [3].

Several separation methods and spectral techniques for the analysis of chiral compounds are available including high performance liquid chromatography (HPLC) [4], gas chromatography (GC) [5], thin layer chromatography (TLC) [6], supercritical fluid chromatography (SFC) [7], nuclear magnetic resonance spectroscopy (NMR) [8], circular dichroism spectroscopy or X-ray crystallography [9]. HPLC is the first-choice technique in the routine analysis of chiral pharmaceuticals; however, CE proved to be in the last decades a viable alternative and a complementary method. There are a series of advantages of using CE in the enantioseparation of optically active pharmaceuticals, related to the rapid method development, high enantioresolution efficiency, low consumption of reagents, analytes, and chiral selectors (CSs), large variety of CSs which can be used and relatively low operational costs. Also, CE is known for its “green” features by comparison with the more frequently used HPLC, as the use of organic solvents is minimum. Furthermore, in CE almost always a direct separation approach is used by simply adding the appropriate CSs into the background electrolyte (BGE) [10,11]. Another advantage is represented by the fact that in CE the migration order of the enantiomers can be reversed based on the nature and characteristics of the CSs, enabling a better understanding and a more practical use of the enantioseparation process [12].

In CE, several types of CSs can be applied (cyclodextrins (CDs), macrocyclic antibiotics, crown ethers, proteins, chiral surfactants, chiral ion pair reagents and other miscellaneous CSs), but by far the most efficient and frequently used are the CD derivatives. Many native and derivatized, neutral and ionized CDs were used successfully during the years for the enantioseparation of a broad spectrum of pharmaceuticals. The advantages of using CDs as CSs in CE are related to their commercial availability, UV transparency, relatively good solubility in aqueous BGEs, and stability over a wide pH range [13].

CDs are oligosaccharides which form a cone-shaped cavity with 6, 7, or 8 glucopyranose units for α-, β- and γ-CD, respectively. Chiral recognition by CDs is generally driven by inclusion complexation, and by secondary interactions involving H-bonds and dipole-dipole interactions with spatially oriented hydroxyl groups and other interactions with the asymmetric carbons of the glucopyranose units [13,14]. It is worth to be mentioned that the formation of external type complexes has been also described [15].

One of the inherent advantages of CE methods over chromatographic methods is the fact that CSs can possess their own electrophoretic mobilities and thus several separation schemes can be experimented. Addition of charged groups (carboxyl, sulfonic acid, sulfobutyl) to the basic CD structure allows the separation of neutral analytes. Furthermore, using oppositely charged CDs to the analytes generates counter mobility, and this allows the use of extremely low CS concentration [14,16].

However, the use of a single CD is not always enough for the enantioseparation of some pharmaceuticals. In some cases, enantioresolution can be improved using a dual CD system, consisting usually of a neutral CD (native, derivatized) combined with a charged one (anionic, cationic, amphoteric).

When dual CD systems are used often there are differences in the complexation mechanisms of the two CDs with the enantiomers of the chiral analyte, which are translated into differences between complexation stabilities, chiral identification patterns, and influence on the electrophoretic mobility of the analytes [17].

When separation occurs because of the difference in the complexation constants, enantiomeric separation can be achieved when the mobilities of the complexed and free form of the analyte differ. This situation in the presence of a single CS system is described in the equation below [18]:

(1) $$\Delta \mu =\frac{\left(\mu_{f}-\mu_{c}\right)\left(K_{R}-K_{S}\right)\left[CD\right]}{1+\left(K_{R}+K_{S}\right)\left[C\right]+K_{R}K_{S}{\left[CD\right]}^{2}}$$

where μ*_f_* and μ*_c_* are the electrophoretic mobilities of the free and complexed forms of the analyte, *K_R_* and *K_S_* are the binding complexation constants of the enantiomers *R* and *S*, and [*CD*] is the CD concentration.

In the case of dual CD systems, both CDs can function synergistically. An interesting approach was developed by Fillet et al. to predict enantioseparation when using dual CDs system through a mathematical model. However, this approach is valid only when complexation with the two CDs are independent (excluding mixed complexes) and the analyte: CD complexation ratio is 1:1. The model can be applied for both neutral and charged analytes and CDs [19].

The following equation for the apparent electrophoretic mobility of the enantiomers in dual CD systems is more appropriate [12]:

(2) $$\mu =\frac{\left(\mu_{f}+\mu_{c1}K_{c1}\left[CD1\right]+\mu_{c2}K_{c2}\left[CD2\right]\right)}{1+K_{c1}\left[CD1\right]+K_{c2}\left[CD2\right]}$$

where, μ is the electrophoretic mobility of the enantiomer, μ*_f_* is the electrophoretic mobility of the free form, μ*_c_*_1_ and μ*_c_*_2_ are the electrophoretic mobilities of the analyte-CD complexes, *K_c_*_1_ and *K_c_*_2_ are the complexation constants of the enantiomer with CD1 and CD2 and [*CD*1] and [*CD*2] are the concentration for the two CDs.

Using this kind of equations can be useful because they can predict changes in the selectivity obtained when using dual CD systems. A more detailed discussion on the chiral separation mechanisms in single and dual CD systems can be found in specific review articles published by Dubský et al. [20] and Chankvetadze [21].

The use of a dual CD system assumes optimization of the affinity pattern by selecting the optimum CS for the enantioseparation and of the mobility terms by selecting the optimum concentration of the charged or uncharged CD. An improvement in enantioresolution can be obtained when one CD accelerates the migration of the analytes and the other decelerates it; or sometimes when the affinity pattern of the enantiomers for each CD is different [22].

The current review focuses on the use of dual CD systems for the analysis of pharmaceuticals and particular features of CD mixture use in CE, presenting specific applications of in the enantioseparation of analytes of interest from different matrices.

## 2. Applications of Dual CD Systems in the Analysis of Pharmaceuticals

The first application of dual CD systems in the separation of enantiomers was published in 1994 by Lurie et al. In this study chiral separation of a high number of basic analytes of forensic interest was resolved by CE using single CDs and mixtures of neutral and anionic CDs. A dual CD system containing heptakis(2,6-di-O-methyl)-β-CD (DM-β-CD) and sulfobutyl ether β-CD (SBE-β-CD) proved to be useful for the simultaneous enantioseparation of illicit drugs (amphetamine, methamphetamine, methcathinone, propoxyphene) using a Tris-phosphate BGE at pH 2.45. When using only a single CD some coelutions happened, however using a dual CD all primary amines were separated. This first application has already shown one of the huge benefits of dual CD systems when the goal is the simultaneous separation of enantiomers of several substances. Chiral resolution and migration times of the enantiomers were controlled by adjusting the ratio of the two CDs; the anionic CD playing the role of counter migrating complexing reagent, while the neutral CD ensured enantiorecognition. An initial theoretical model was developed to characterize the impact of neural and anionic CDs on the chiral resolution of cationic analytes. However, the addition of SBE-β-CD resulted in increased tailing and migration times when compared with the use of single DM-β-CD, probably due to electrodispersion [23].

In another study by Anigbogu et al. the enantiomers of aminoglutethimide were separated in a basic BGE at pH 9.0 using a mixture of neutral β-CD and anionic carboxymethyl-β-CD (CM-β-CD). At this pH, aminoglutethimide is neutral and no chiral interactions were observed when using the CDs individually. Apparently the neutral β-CD provided enantioselectivity, while the anionic CM-β-CD provided a differential migration rate. The basic pH of the optimized selected method resulted in a strong electroosmotic flow (EOF), which was accompanied by a short analysis time. The method was compared with a micellar electrokinetic chromatography method (MEKC) at the same pH using only β-CD as CS which resulted only in partial resolution of the enantiomers and a capillary zone electrophoresis (CZE) technique at acidic pH (3.0) where small chiral interactions were observed with neutral α- and γ-CD [24].

Lelièvre et al. developed a CE chiral separation method for arylpropionic acid non-steroidal anti-inflammatory drug (NSAID) derivatives (carprofen, flurbiprofen, ketoprofen, naproxen, suprofen) using a CD mixture composed of a cationic CD, amino-β-cyclodextrin (NH_2_-β-CD) and a neutral one, trimethyl-β-CD (TM-β-CD) in an acidic BGE at pH 2.3. In this case, interactions with NH_2_-β-CD, are not stereoselective, however provide the differential migration rates of the enantiomers, while TM-β -CD allows the chiral recognition. Also, a theoretical model regarding selectivity was developed for dual CDs separation systems, based on the concept of the apparent constant of complex formation [25].

In another study, regarding chiral NSAIDs (carprofen, fenoprofen, flurbiprofen, ibuprofen, indoprofen, ketoprofen, sulindac, surprofen, tiaprofenic acid) Fillet et al. obtained the baseline enantioseparation by CE using a dual CD selector system containing neutral TM-β-CD and the anionic SBE-β-CD at pH 3.0. In an acidic BGE these analytes are almost neutral, consequently, the use of uncharged CDs alone is inefficient, while the use of charged CDs gave some chiral resolution, which was significantly improved when the dual CD system was applied. In this system the charged CD played the role of the carrier giving the analytes an apparent electrophoretic mobility, while the uncharged CD provided enantioselectivity. Triethanolamine was used as BGE additive to provide a weak EOF; as the anionic CD moved with EOF; remarkably high resolution and relatively short analysis time (less than 15 min) for a chiral separation were obtained [26].

Based on the comparison of the two previously mentioned studies we can observe that aryl propionic acid derivative NSAIDs in a mixture can be separated at acidic pH using a dual CD system. One of the CD should be charged, as both cationic and anionic derivatives were applied to play the carrier role, and the other CD is neutral and is used to improve selectivity.

In a follow-up study, the utility of the previously developed CD system was verified for the enantioseparation of weak acids and neutral compounds (chlormezanone, chlorthalidone, hexobarbital, mephenytoin, mephobarbital, pentobarbital, secobarbital, thiopental). Systems containing DM-β-CD and TM-β-CD as neutral CDs and CM- β-CD and SBE-β-CD as anionic ones were tested at different pH values. All the analytes were baseline enantioseparated at pH 3.0 except for the anxiolytic drug chlormezanone, which was resolved at pH 5.0 using a combination between TM-β-CD and CM-β-CD. Higher resolutions were obtained at pH 5.0 for all the analytes; at this pH, the results obtained when using CM-β-CD as anionic CD were better than in the case of SBE- β-CD, due to the substantial increase in the negative charge of CM-β-CD, because of pH increase from 3.0 to 5.0 [27].

In another study by Fillet et al. the enantioseparation selectivity in dual and single CD CS systems was compared using basic drugs (brompheniramine, chlorpheniramine, dimethindene, ephedrine, verapamil) as model substances. Different CD combinations were tested: CDs with opposite chiral recognition pattern but with the same effect on analyte mobility (CM-β-CD and SBE-β-CD) and CDs with the same chiral recognition pattern but with opposite effect on analyte mobility (DM-β-CD and TM β-CD). The use of a CD system in which the CSs have the same effect on the mobility of the analytes, decreased separation efficiency, while the use of a CD system in which the CSs have opposite effect on the mobility of the analytes seem to be beneficial for the separation [28].

It can be seen, that as well as in the case of acidic character compounds, basic drugs can be separated using a dual anionic-neutral CD system. However, in contrast to acidic drugs, basic drugs have their own mobility at acidic pH. Moreover, combination of methylated CD with CM- β-CD or SBE- β-CD seem to be the most effective dual system.

CE with a dual CD chiral system was applied by Fillet et al. for the enantioselective determination of *S*-naproxen in tablets. Several single and dual CD systems were applied in a phosphoric acid-triethanolamine BGE at pH 3.0, the best results were obtained when using a mixture of TM-β-CD and SBE-β-CD, similar with the one used previously by the same research group for the enantioseparation of other NSAIDs [24]. The method was validated for the determination of enantiomeric impurity, *R*-naproxen, in a 0.1–2% range. This method proves the applicability of dual CD systems for the verification of enantiomeric purity of analytes used as pure enantiomers [29].

Meyrig et al. developed a CE method for the enantioseparation of thalidomide and three of its hydroxylated metabolites using a dual CD system composed of native β-CD and anionic SBE-β-CD and a polyacrylamide-coated capillary. The separation of thalidomide and its hydroxylated metabolites by CE was verified in a previous study by the same research group using four charged CDs, and their combinations with β-CD, the best results being obtained when using CM-β-CD, however the simultaneous baseline separation of all the enantiomers was not achieved [30]. In the dual system, SBE-β-CD exhibited enantioselectivity towards the analytes, while β-CD enhances enantioselectivity of recognition in the system. The method was used to characterize biotransformation of thalidomide in vitro by rat liver microsomes. The CE method of the incubated samples together with X-ray diffraction was used for the determination of absolute configuration of metabolites and to characterize the stereoselective biotransformation processes. The results support preferential metabolic pathways for *R*- and *S*-thalidomide [31]. The use of the method was further extended to one additional metabolite, using the same combination of β-CD and SBE-β-CD, carrier mode in which CDs transport the neutral compounds to the detector and polyacrylamide-coated capillary; using this condition the simultaneous separation of all eight enantiomers was resolved. These studies are a good example regarding the bioanalytical application of dual CD systems [32].

In a study published by Abushoffa et al. a single isomer anionic heptakis-6-sulfato-β-cyclodextrin (HS-β-CD) was used in combination with neutral TM-β-CD for the CE enantioseparation of arylpropionic acid NSAIDs (fenoprofen, flurbiprofen, ibuprofen, ketoprofen) using a pH 2.5 phosphoric acid–triethanolamine BGE in the reversed polarity mode. The model compounds are acidic and are not ionized at pH 2.5, the use of HS-β-CD as a single CS gave poor resolution, however in dual CD system HS-β-CD provided the analytes with the suitable mobility while TM-β-CD provided enantioselectivity. Affinity constants for the enantiomers with the both CDs were determined, using linear regression in a two-step approach; for HS-β-CD were calculated in single CD systems while with TM-β-CD were calculated in dual systems. A mathematical model was developed to predict the best analytical conditions for the CE separation of aryl propionic derivatives in HS-β-CD—TM-β-CD dual systems [33].

The same research group applied the use of a mixture of charged CDs for the enantioseparation of the four analytes from the previous study. A combination of two oppositely charged CD derivatives were used; a single isomer cationic CD, permethyl-6-monoamino-6-monodeoxy-β-CD (PMMA-β-CD) and an anionic CD, HS-β-CD in a phosphoric acid-triethanolamine BGE at pH 2.5. Significant improvement in selectivity and resolution was observed, as the two CDs lead independent enantioselective complexation with the analyte enantiomers and exhibit not only opposite effects on the electrophoretic mobility of the analytes but also opposite affinity patterns towards the enantiomers. Binding constants were determined using linear regression method, to predict conditions, giving rise to high selectivity and resolution using the previously developed mathematical models. The use of single isomer CDs allows prediction of the analytical performance (resolution, mobilities) of the method. However, it should also be noted, that these derivatives are much more expensive, and they separation capacity does not exceed the one of randomly substituted CD derivatives [34].

Pérez-Maseda et al. developed a CE enantioseparation method for a potential novel NSAID cyclooxygenase-2 (COX-2) inhibitor. The eutomer of this compound is the *S*-enantiomer (E-6232), while the *R*-enantiomer (E-6231) was considered a chiral impurity; other five degradation impurities were alsoanalyzed. A mixture of DM-β-CD and SBE-β-CD was used in the chiral separation, the limit of detection (LOD) and limit of quantitation (LOQ) was 0.03% and 0.1% of distomer. The use of single SBE-β-CD proved to be also efficient, however the dual CD system offered advantages in terms of higher enantioresolution and sensitivity [35].

Tábi et al. developed a CE enantioseparation method for deprenyl, an irreversible monoamine oxidase enzyme (MAO) inhibitor and eight of its metabolites, including the active metabolite, *N*-deprenyl oxide. The enantioresolution of all analytes was achieved by using a dual CD system containing DM-β-CD in combination with CM-β-CD in a Tris-phosphate BGE at pH 2.7. The method was applied for the determination of deprenyl metabolites in rat urine; stereoselective metabolism of deprenyl was confirmed. This method proves that CE with dual CD selector system could be an attractive tool for detect and separate chiral metabolites in biological fluids [36].

Lin et al. developed a MEKC enantioseparation method for miconazole, an antifungal agent with an imidazole structure, using a borate-sodium dodecyl sulphate (SDS) BGE at pH 9.5 and a mixture of mono-3-*O*-phenylcarbamoyl-β-CD (MPC-β-CD) and β-CD as CS. The separation resolution significantly increased using the dual CD system when compared with the individual CDs alone [37].

An enantioselective method for the verification of chiral purity of a potential antianginal agent with a benzoaxathiepin structure was developed by Beaufor et al. The substance has two chiral centers, consequently four optical isomers, however the eutomer is considered to be the *R*,*S*-enantiomer, while the *R*,*R*-, *S*,*S*- and *S*,*R*-forms are considered to be chiral impurities. The combination of hydroxypropyl-γ-CD (HP-γ-CD) and CM-β-CD was used in the separation added in a phosphate BGE at pH 6.4 [38].

The enantiomeric purity of a basic drug efaroxan, a selective α-2-adrenoreceptor antagonist, was verified by CE using a dual CD system. The method published by Lorin et al. uses a mixture of DM-β-CD and CM-β-CD added in a phosphoric acid–triethanolamine BGE at pH 3.0. The method was validated for the enantiomeric impurity determination of the distomer *S*-efaroxan at 0.05% level [39].

Chu et al. developed a CE enantioselective analysis method for chiral separation of the antiparkinsonian drug rotigotone, a non-ergolinic dopamine receptor agonist and two related chiral impurities. A mixture of methyl-β-CD (M-β-CD) and sulfated-β-CD (S-β-CD) was added in a phosphate buffer at pH 2.5. The LOD and LOQ were 0.003 and 0.01 mM for each enantiomer, respectively [40].

**Figure 1:**
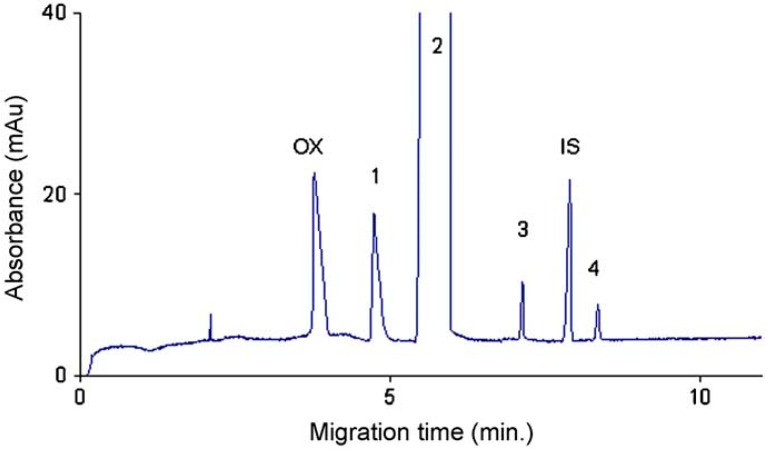
Electropherogram of 5 mg/mL S-CIT oxalate containing approximately 2.4% R-CIT and 0.1% of citadiol enantiomers (1—*R*-CIT, 2—*S*-CIT, 3—*S*-citadiol, 4—*R*-citadiol, OX—acid oxalic, IS—internal standard (salicylic acid); experimental conditions: 20 mM phosphate BGE, pH 2.5, CS 22 mg/mL S-β-CD + 0.5 mg/mL β-CD, −20 kV voltage; 28 °C temperature; 50 mbar/5 s injection, detection at 205 nm) Reprinted from Sungthong et al. [41] with permission from Elsevier.

Sungthong et al. developed a stereospecific CE method for the simultaneous determination of optical purity and other related compounds of *S*-citalopram, a selective serotonin reuptake inhibitor (SSRI) antidepressant. Citalopram is used in therapy both as racemic mixture and in the form of its eutomer, *S*-citalopram. A phosphate BGE at pH 2.5 containing a mixture of native β-CD and S-β-CD was applied in the enantioseparation. For method optimization a central composite face-centered factorial design was used. During the optimization, the concentration of β-CD was kept constant at 0.5 mg/mL as preliminary experiments revealed not significant effect in the range of 0.5–2 mg/mL; and the effect buffer concentration, applied voltage, temperature and S β-CD concentration were studied. The method was validated for the determination of the distomer, *R*-CIT and the enantiomers of an impurity, citadiol. LOD was 0.02%, while LOQ 0.05% for all compounds reported to a 5 mg/mL concentration of *S*-CIT. The method was applied for the determination of chiral purity of *S*-CIT in bulk substance and tablets. A representative electropherogram for the determination of *S*-CIT chiral impurities is presented in Figure 1 [41].

A CE chiral separation method for the enantioseparation of three glitazone derivatives (balaglitazone, pioglitazone, rosiglitazone) was developed by Jamali et al. Initially enantiorecognition of DM-β-CD and SBE-β-CD was investigated in single CD systems at different pH values. The baseline separation of all analytes was achieved when using a dual CD system composed of the two CDs added to a sodium tetraborate BGE ay pH 9.70. Optimization of analytical parameters was made using factorial design experiment. ^1^H-NMR studies were performed to characterize the CD-analyte interactions; the results showed that SBE-β-CD interacts primarily with the basic part of glitazone derivatives while DM-β-CD interacts with the phenyl ring [42].

A CE method was used for the simultaneous determination of impurities of dexamphetamine, including levoamphetamine and related substances (1*S*,2*S*)-norpseudoephedrine, (1*R*,2*S*)-norephedrine, phenylacetone and phenylacetone oxime. The method developed by Wongwan et al. used a phosphate BGE at pH 3.0 and a combination of SBE-β-CD and S-β-CD as a dual CD selector system. The effect of the degree of substitution of SBE-β-CD was investigated. The LOD values were between 0.01–0.02%. This study proves the applicability of dual CD systems containing two anionic CD in the enantioseparation by CE [43].

Lipka et al. worked on the CE enantioseparation of six agonists and antagonists with tetrahydronaphthalenic structures for the melatonin (*N*-acetyl-5-methoxytryptamin) binding site. The baseline separation was successful using polyethylene oxide dynamically coated capillaries and a dual CD selector system. The composition and concentration of the CD system were optimized systematically. The best results were obtained with a mixture of γ-CD and highly sulfated-β-CD (HS-β-CD) in a phosphate buffer at pH 2.5 [44].

A dual CD system composed of 6-monodeoxy-6-mono(3-hydroxy)-propylamino-β-cyclodextrin (HPA-β-CD) and DM-β-CD was used by Wagner et al. for the determination of excitatory amino acids in brain samples by CE using laser induced fluorescence (LIF) detection. The method was used to separate aspartate and glutamate enantiomers to investigate the putative neuromodulator function of D-aspartate and D-glucose in the central nervous system (CNS). 4-Fluoro-7-nitro-2,1,3-benzoxadiazole was used as fluorescent derivatization agent. The method was applied to analyze brain samples of 1-day-old chickens [45].

Sohajda et al. developed a CE method for the enantioseparation of imperanene, a polyphenolic compound extracted from the plant *Imperata cylindrica* using CDs as CS. A complex CD screening involving 27 CDs was performed to establish the optimum CS. The best result was obtained using a dual CD system containing 6-monodeoxy-6-mono-(3-hydroxy)-propylamino-β CD (MPA-β-CD) and sulfobutyl-ether-γ-CD (SBE-γ-CD) added to a borate BGE at pH 9.0. The average stoichiometry of the complex was determined with Job’s method using NMR-titration, the results showed a 1:1 complex for both enantiomers. One of the curiosities of this study is that the best result was obtained using a dual system containing CDs with opposite charge based on a complex CD screening [46].

Wan Ibrahim et al. developed a MEKC method for the simultaneous determination of three imidazole antifungal drugs (fenticonazole, isoconazole, tioconazole) in a mixture. The baseline separation was obtained using a mixture of two neutral CD, HP-γ-CD and DM-β-CD added to a phosphate- SDS BGE at pH 7.0. The use of single CDs gave an incomplete separation, however the mixture of the two neutral CD derivatives was able two separate the six stereoisomers. LOD values for the three analytes ranged from 2.70–7.70 mg/L. The method was applied for the determination of the three drugs in spiked human urine and from pharmaceutical preparations [47].

Separation of five agomelatine analogues, with potential antidepressant effect, was achieved by Lipka et al. using dynamically coated capillary with polyethylene oxide and a dual CD system as CS. The best results were obtained using a phosphate BGE at pH 2.5 and a mixture of 6-monodeoxy-6-monoamino-β-CD (MMA-β-CD) and sulfated-γ-CD (S-γ-CD) [48]. Like in previously mentioned study by Sohajda et al. [46], in this study a dual CD system containing positively and negatively charged CDs at acidic pH and coated capillary was used to avoid the possible interaction between the CDs and the capillary wall.

Yu et al. verified the applicability of a newly synthetized cationic CD, single isomer mono-6-deoxy-6-piperdine-β-cyclodextrin (PIP-β-CD), for the enantioseparation by CE of meptanizol, an opioid-type analgesic with mixed agonist–antagonist effects and three of its intermediates. To enhance chiral resolution a dual CD system containing β-CD and PIP-β-CD was applied. This example shows the advantage of using dual CD system in the separation of molecules with more chiral centers [49].

**Figure 2:**
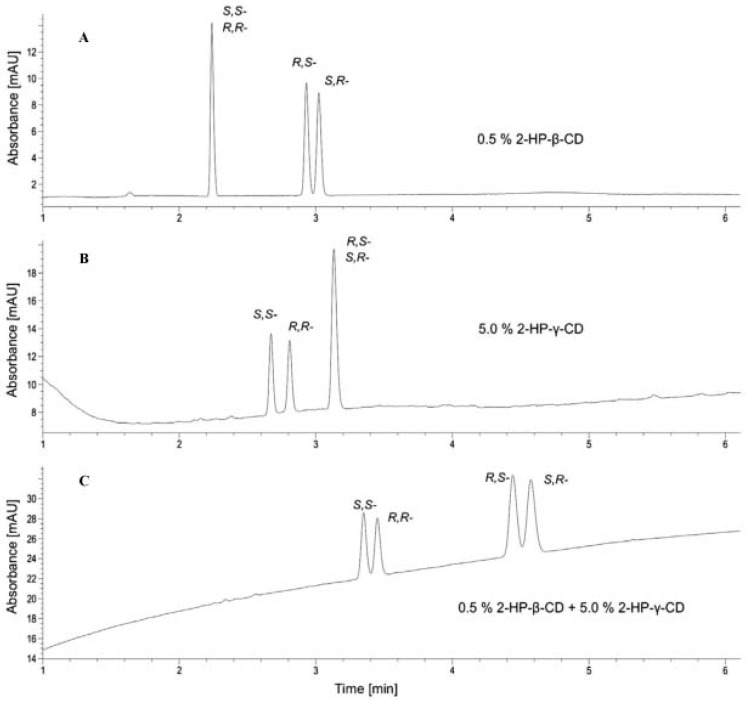
Chiral separation of tapentadol enantiomers by CE in single and dual CD systems ((**A**)—CS 0.5% HP-β-CD; (**B**)—CS 5% HP-γ-CD; (**C**)—CS 0.5% HP-β-CD + 5% HP-γ-CD; experimental conditions: 50 mM phosphate BGE, pH 2.5, −25 kV voltage; 15 °C temperature; 25 mbar/5 s injection, detection at 210 nm) Reprinted from Znaleziona et al. [50] with permission from Elsevier.

The chiral separation of the four stereoisomers of tapentadol, a centrally acting analgesic agent, was described by Znaleziona et al. The best results were obtained when using a mixture of two neutral CDs, hydroxypropyl-β-CD (HP-β-CD) and HP-γ-CD added to a borate BGE at pH 9.5. HP-β-CD resolved the separation of the *S,R*- and *R,S*-enantiomers, HP-γ-CD resolved the separation of the *S*,*S*- and *R*,*R*-enantiomers; while the use of the dual CD system separated all four stereoisomers. The influence of single and dual CD on the enantioseparation of the tapentadol four stereoisomers is presented in Figure 2 [50].

Melani et al. developed a CE method for the chiral separation of sulpiride, an atypical antipsychotic drug with benzamide structure. Previously the use of a Quality by Design (QbD) strategy was reported by Orlandini et al. for the chiral purity study of the eutomer levosulpiride (*S*-sulpiride) by CE using a dual CD system composed of M-β-CD and S-β-CD added in a Britton-Robinson BGE at pH 3.45 [51]. Molecular modelling was used to explain affinity of sulpiride enantiomers towards the two CDs, which were used in the previous study. Two-dimensional rotating-frame nuclear Overhauser effect spectroscopy (2-D ROESY-NMR) experiments were applied to characterize complex formation; the results indicated the inclusion of the benzene sulfonamide moiety of sulpiride in the hydrophobic cavity of the CDs [52].

Szabó et al. developed a CE method for the simultaneous determination of four H1 antihistamine derivatives (brompheniramine, chlorpheniramine, cetirizine, promethazine). After an initial CD screening the use of a dual CD system composed of β-CD and SBE-β-CD added in a phosphate BGE at pH 7.0 was selected as the optimum solution. The two selected CDs showed synergic effect on enantioseparation; resulting in the simultaneous baseline separation of all 8 enantiomers in a short analysis time (less than 8 min) with good chiral resolution. Even if chemically related drugs from the same therapeutic class are usually not administered together, the development of generic methods for their simultaneous determination became a tendency in recent years, as it can be applied for the determination of any of these analytes without the need to develop separate methods for each analyte. The method was validated and used for the determination of levocetirizine from tablets [53].

Nováková et al. reported a CE method for the enantioseparation of leucine and phenylalanine benzothiazole derivatives, with potential antimicrobial effects. Dual CD system consisting of two neutral CDs; β-CD with HP-β-CD for the leucine derivative and β-CD with HP-γ-CD for the phenylalanine derivative, added in a phosphate BGE at pH 3.5, proved to be the best solution for the enatioseparation [54].

Delplanques et al. developed a CE method using single and dual CD systems for the enantiomeric and diasteromeric separation of dihydropyridine analogues, with one or two chiral centres. Ten dual CD systems proved to be successful in the separation, among the tested CDs, HS-γ-CD and SBE-β-CD proved to be the most efficient selectors. This work underlines the challenges analysts must overcome in developing chiral separations as there is a large diversity of CDs used as CSs which can be mixed in multiple combinations [55].

The chiral separation method for ketamine metabolites, norketamine, hydroxynorketamine and 5,6-dehydronorketamine by CE was published by Sandbaumhüter et al. The best results were obtained using a mixture of CDs composed of S-β-CD and HS-γ-CD added to a phosphate BGE at pH 3.0. A liquid-liquid extraction (LLE) was used for sample preparation. The method was applied to analyze plasma samples from dogs and horses after i.v. ketamine administration and in an in vitro study in which ketamine was incubated with equine liver microsome [56].

Papp et al. developed a CE method for the enantioseparation of two proton pump inhibitors (PPI) (lansoprazole, rabeprazole). Single and dual CD systems were screened in a preliminary analysis. A mixture containing γ-CD and SBE-β-CD was selected and added in a phosphate BGE at pH 7.0. An experimental design approach was used for method optimization, a fractional factorial screening design followed by a central composite design. The optimum ratio of the two CDs was different for lansoprazole and rabeprazole, respectively. The method was validated and applied for the determination of distomers (*S*-lansoprazole, *S*-rabeprazole) as chiral impurities in dexlansoprazole and dexrabeprazole samples [57].

**Figure 3:**
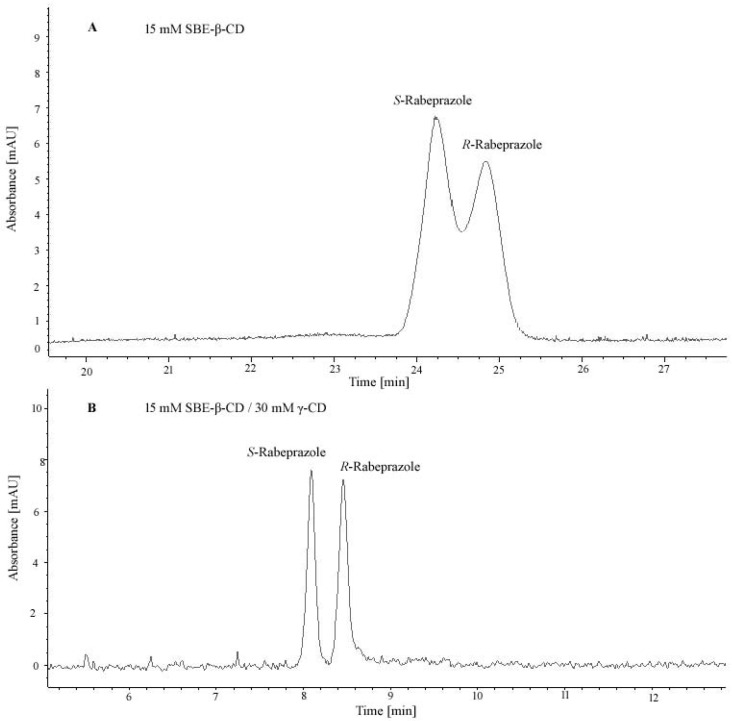
Chiral separation of rabeprazole enantiomers by CE in single and dual CD systems ((**A**)—CS 15 mM SBE-β-CD, (**B**)—CS 15 mM SBE-β-CD/30 mM γ-CD; experimental conditions: 25 mM phosphate BGE, pH = 7.0, +20 kV voltage; 18 °C temperature; 50 mbar/3 s injection, detection at 210 nm).

A representative electropherogram from our own collection which illustrates the increase of chiral resolution and decrease of analysis time using a dual CD system composed of γ-CD and SBE-β-CD in the case of rabeprazole is presented in Figure 3.

Harnisch et al. published a CE method for the simultaneous determination of chiral and achiral purity of *S*-dapoxetine, a selective serotonin reuptake inhibitor (SSRI), regarding the distomer *R*-dapoxetine and structurally related impurities. For the optimization of the analytical method an initial fractional factorial design was applied followed by face centered central composite design. The optimum CD CS system contained DM-β-CD and S-γ-CD added to a phosphate BGE at pH 6.3. The method was validated and applied for the analysis of dapoxetine in tablets [58].

The same methodology was applied by Krait et al. for the determination of chiral purity of dextromethorphan, a centrally active antitussive drug, regarding its distomer, levometorphan. A dual CD system containing M-β-CD and S-β-CD added in a phosphate BGE at pH 6.5 was used. An initial fractional factorial design followed by a face centered central composite design was used for method optimization. The method was validated and applied for the analysis of dextromethorphan in capsules [59].

Casado et al. applied CE method with a dual CD system for the simultaneous enantioseparation of six phenoxy acid herbicides from a complex mixture. The CD CS system contained two neutral derivatives HP-β CD and TM-β-CD added in a phosphate BGE at pH 7.0. The method development was based on a previously published theoretical model proposed by Dubský et al., which permits prediction of what happens in a dual CD system by performing individual measurements with each CD individually at different concentration levels [60]. The results confirmed the validity of the theoretical model, improving enantiomeric resolution and reducing migration times of the analytes, enabling to establish the most favorable proportion between the two CDs. Additionally, the apparent complexation constants between enantiomers and both CDs were calculated [61].

The previously described methods analytical conditions are summarized in Table 1.

## 3. Discussion

To choose an efficient dual CD system, and explain chiral separation, analysts must consider both chiral interactions which occurs at molecular level and chiral recognition which leads to chiral discrimination. Consequently, both mobility and affinity effects must be taken into consideration and an explanation about affinity patterns that generates increased enantioseparation may be drawn depending on the effect of both CSs on the electrophoretic mobility of the analyte enantiomers.

In some situations, an increase in the selectivity of enantioresolution can be observed when changing from a single CD CS system to a dual CD system. Also, sometimes, the intrinsic chiral separation mechanism can be better understood when a dual CD system is applied as it is possible to define selectivity whether complexing agents are considered separately or all together.

Several combinations of CDs can be used in practice: combinations between a neutral CD with a charged one, between two charged CDs with the same charge or between two neutral CDs [13,14,16].

Analyte-CD complexation is the main trigger that leads to enantiomer separation by CE when this leads to differences in mobility between the free and complexed formed of the analytes and stability differences between the complexes formed by the CD with the enantiomers of the analyte. In the case of charged CDs, they have their own electrophoretic mobility, and can be used as carriers for the separation of neutral and ionizable analytes in uncharged forms [17,19].

In some dual CD systems, the chiral discrimination capacity of one CD can lead to enantioresolution only in the presence of the second CD. For example, if we have an uncharged chiral analyte, its enantiomers can exhibit chiral interaction with a neutral CD without significant enantioresolution because of the small mobility differences between the free and the complexed forms. When a charged CD is added to the BGE, it can provide an apparent electrophoretic mobility to the analyte, which increases enantioresolution; even if the interactions with the charged CD are not enantioselective [17,19,62].

In other dual CD systems, the interactions with the two CDs can be both enantioselective, and have opposite effects on the migration of enantiomers, one CD accelerates while the other decelerates them, in this case enantioselectivity is obtained if the affinity patterns of the enantiomers towards the CDs are different. The same condition must be met, if one of the CDs has little effect on the electrophoretic mobility of the analyte, as in the case of an uncharged analyte and a neutral CD. A mixture of two CDs exhibiting the same effect on analyte mobility, as both CDs accelerate or decelerate migration of the enantiomers, seem to be inefficient in the separation [17,19].

But in another hand, the variations in chiral selectivity observed when using dual CD systems are not always explainable; in some situations, the two CDs can have synergistic or antagonistic effects on the chiral resolution, which makes the overall process unpredictable.

To obtain information about complex stoichiometry, binding constants, stereoselectivity or intermolecular analyte-CD interactions other instrumental techniques such as NMR, MS or X-ray crystallography can be used. Molecular modelling can also be a valuable tool in establishing analyte-CD complex structure and mechanism of enantiorecognition [63,64].

Initially a trial-error strategy was applied in choosing the optimum CD for the separation, however in the past 20 years the theory enantioseparation in dual CD mixtures has been explained and understood. Theoretical mathematical models have been developed and can be used as feasible practical approaches in chiral method development [22,60,65].

Also, the use of QbD principles and design of experiments (DoE) represents a new strategy for the development of CE separation methods suitable for chiral analysis [51,66]

It is essential that choice of the optimum CD mixture as CSs, should be complemented using the right ratio between the two CD and DoE is an efficient tool to establish this with a minimum number of experiments [57,66,67].

It should be noted that even if in CE enantioseparations usually dual CD systems are used, CD may be combined sometimes also with other CSs (chiral ionic liquid, chiral antibiotic, polysaccharide, crown ethers and others) or rarely combined CS system without CDs can be applied [10,68].

## 4. Conclusions

There is only one review published in literature regarding the specific use of CD dual systems in CE for the analysis of pharmaceuticals, this review was published in 2000 by Fillet et al. [13]. However, the application in the pharmaceutical field of dual CD systems has broadened and separation mechanism involved in chiral discrimination has been more profoundly understood in the past twenty years.

Of course, there are a series of reviews published in the last ten years regarding the application of CDs in general as CSs in CE, reviews in which CD dual system are mentioned as an interesting and useful option. We can mention here the reviews published by Rezanka et al. [13] and Saz et al. [16]. Generalized mathematical models of separation in both single and dual CD systems were proposed by Dubský et al. [60,65,69]. Also, a review regarding theoretical models used to explain separation mechanism, published by Müllerová et al. [22] is worth mention.

It is interesting to mention that initial works involving the use of CD dual system in CE enantioseparations focused mainly on the analysis of model compounds with particular structural and electrophoretic properties in order to develop theoretical models used to characterize the influence of chiral discrimination ability and electrophoretic mobility of the two CDs on the overall selectivity of the enantiomeric separation. As information about separation mechanism in dual CD system was clarified, research direction changed, and studies focused mainly on the application of dual CD systems on the enantiomer separation of specific chiral analytes; the use of dual CD systems for the verification of enantiomeric purity, quantitative analysis from pharmaceutical formulations or pharmacokinetic studies have been reported.

In CE, the right selection of the optimum CD to be used to achieve chiral resolution is the essential step in method development. When the use of a single CD does not provide enantioresolution, the addition of a second CD may generate separation. In the case of dual CD systems usually a combination of an anionic CD (e.g., SBE-β-CD, CM-β-CD, S-β-CD) with a neutral CD (e.g., methylated-β-CD, HP-β-CD) is used. It is a fact that many analytes have basic character at an acidic pH, consequently, are positively charged and will have a higher affinity towards negatively charged CDs, increasing enantiorecognition capability. The use of dual CD systems is extremely useful in the case of analytes in uncharged form; in this situation it is preferable that the charged CDs should have no enantioselectivity towards the analyte or exhibit a chiral recognition pattern opposite to the neutral CD.

Based on the described separation mechanism, the two enantiomers of a chiral analyte under interaction with a dual CD system are likely to differ in their mobilities, compared with single CD systems where the mobilities are likely to be the same. Although generally different distribution constants between the two enantiomers and CDs in single CD system dictates enantioseparation, in dual CD systems additional electrophoretic enantioselective mechanisms resulting from different electrophoretic mobilities of the enantiomer-CD complexes play an important role in separation. Dual CD systems are more selective than single CD systems due to this additional separation mechanism.

The use of dual CDs system can be a useful tool in the enantioseparation of pharmaceuticals, offering good perspectives and high potential, as novel dual systems are continuously developed and applied in diverse analytical fields.

## Figures and Tables

**Table 1:** Electrophoretic conditions for the enantioseparation of drugs using dual cyclodextrin systems.

Electrophoretic Conditions	Chiral Selector	Analyte	Reference
25 mM Tris-phosphoric acid, pH 2.45, 10% methanol, 30 kV, 30 °C, UV 210 nm	5 mM DM-β-CD, 1 mM SBE-β-CD	amphetamine, metamphetamine, methcathinone, propoxyphene—bulk substances	[23]
10 mM phosphate-6 mM borax, pH 9.0, 50% methanol, 10 kV, UV 205 nm	1 mM β-CD, 5 mM CM-β-CD	aminogluthetimide—bulk substance	[24]
34 mM phosphoric acid, pH 2.30, 20 kV, 25 °C, UV 240 nm (calprofen, flurbiprofen), 210 nm (ketoprofen), 230 nm (naproxen), 254 nm (suprofen)	10 mM TM-β-CD, 20 mM NH_2_-β-CD	carprofen, flurbiprofen, ketoprofen, naproxen, suprofen—bulk substances	[25]
100 mM phosphoric acid-triethanolamine, pH 3.0, −25 kV, 25 °C, 230 nm (carprofen, fenoprofen, flurbiprofen), 210 nm (ibuprofen), 280 nm (indoprofen, ketoprofen, sulindac, suprofen, tiaprofenic acid).	15 mM TM-β-CD, 5 mM SBE-β-CD	carprofen, fenoprofen, flurbiprofen, ibuprofen, indoprofen, ketoprofen, sulindac, surprofen, tiaprofenic acid—bulk substances	[26]
100 mM phosphoric acid-triethanolamine, pH 3.0, −25 kV, 25 °C, UV 210 nm 100 mM phosphoric acid-triethanolamine, pH 5.0, −25 kV, 25 °C, UV 210 nm	10 mM TM-β-CD (DM-β-CD), 5 mM SBE-β-CD 10 mM TM-β-CD (DM-β-CD), 10 mM CM-β-CD	chlormezanone, chlorthalidone, hexobarbital, mephenytoin, mephobarbital, pentobarbital, secobarbital, thiopental—bulk substances	[27]
100 mM phosphoric acid, pH 3.0, 25 kV, 25 °C, UV 210 nm	10 mM TM-β-CD, 5 mM CM-β-CD	brompheniramine, chlorpheniramine, dimethindene, ephedrine, verapamil—bulk substances	[28]
100 mM phosphoric acid-triethanolamine, pH 3.0, −25 kV, 25 °C, UV 210 nm	20 mM TM-β-CD, 5 mM SBE-β-CD	naproxen chiral purity—tablets	[29]
5 mM ammonium acetate, pH 4.5, −30 kV, 25 °C, UV 210 nm	12 mg/mL β-CD, 12 mg/mL SBE-β-CD	thalidomide, 3 hydroxylated metabolites—rat liver fractions	[31]
100 mM phosphoric acid-triethanolamine, pH 2.5, −25 kV, 25 °C, UV 214 nm	20 mM TM-β-CD, 3 mM HS-β-CD	fenoprofen, flurbiprofen, ibuprofen, ketoprofen—bulk substances	[33]
100 mM phosphoric acid-triethanolamine, pH 2.5, 25 kV, 25 °C, UV 214 nm	4 mM HS-β-CD, 18 mM PMMA--β-CD	fenoprofen, flurbiprofen, ibuprofen, ketoprofen—bulk substances	[34]
50 mM sodium tetraborate, 30% methanol, pH 9.2, 30 kV, 20 °C, UV 315 nm	0.5 mM DM-β-CD, 7.1 mM SBE- β-CD	COX-2 inhibitor, chiral impurities—bulk substances	[35]
20 mM Tris-phosphate, pH 2.7, 25 kV, 20 °C, UV 214 nm	9 mM DM-β-CD, 0,5% CM-β-CD	deprenyl, deprenyl metabolites—rat urine	[36]
50 mM sodium tetraborate, 50 mM SDS, 1 M urea, pH 9.5, UV 214 nm	15 mM β-CD, 15 mM MPC- β-CD	miconazole—bulk substance	[37]
30 mM phosphate, pH 6.4, 30 kV, 20 °C, UV 214 nm	10 mM HP-γ-CD, 10 mM CM- β-CD	benzoaxathiepin antianginal agent, chiral impurities—bulk substances	[38]
100 mM phosphoric acid-triethanolamine, pH 3.0, 20 kV, 25 °C, UV 214 nm	7,5 mM DM- β-CD, 3 mM CM-β-CD	efaroxan, chiral impurity—bulk substance	[39]
100 mM sodium phosphate buffer, pH 2.5, −20 kV, 20 °C, UV 200 nm	2% (*w*/*v*) M-β-CD, 2% (*w*/*v*) S-β-CD	rotigotone, chiral impurities—bulk substances	[40]
20 mM phosphate, pH 2.5, −20 kV, 28 °C, UV 205 nm	0.5 mg/mL β-CD, 22 mg/mL S-β-CD	S-citalopram—chiral purity—bulk substance, tablets	[41]
50 mM sodium tetraborate, pH 9.7, 15 kV, 30 °C, UV 200 nm	3 mM DM-β-CD, 12 mM SBE-β-CD	balaglitazone, pioglitazone, rosiglitazone—bulk substance	[42]
50 mM phosphate, pH 3.0, −10 kV, 20 °C, UV 200 nm	80 mg/mL SBE-β-CD, 25 mg/mL S- β-CD	dexamphetamine -chiral purity—bulk substances	[43]
25 mM phosphoric acid-triethanolamine, pH 2.5, 25 kV, 20 °C, UV 190 nm	10 mM γ-CD, 1.5% (*w*/*v*) HS-β-CD	agonist and antagonist melatoninergic ligands—bulk substances	[44]
75 mM sodium tetraborate, pH 9.0, 30 kV, 20 °C, UV 210 nm	10 mM MPA- β-CD, 12.5 mM SBE-γ-CD	imperanene	[46]
35 mM phosphate, 50 mM SDS, pH 7.0, 15% (*v*/*v*) acetonitrile, 27 kV, 20 °C, UV 210 nm	35 mM HP-γ-CD, 10 mM DM-β-CD	fenticonazole, isoconazole, tioconazole—spiked urine sample, pharmaceutical preparation	[47]
25 mm phosphate, pH 2.5, 25 kV, 25 °C, UV 227 nm	10 mM MMA-β-CD, 5% (*w*/*v*) S-γ-CD	agomelatine analogues—bulk substances	[48]
20 mM phosphate, pH 3.0, 20 kV, 20 °C, UV 237, 271 nm	40 mM HP-β-CD, 5 mM PIP-β-CD	meptanizol, 4 intermediates—bulk substances	[49]
50 mM phosphate, pH 2.5, 25 kV, 15 °C, UV 210 nm	0.1% (*w*/*v*) HP-β-CD, 0.5% (*w*/*v*) HP-γ-CD	tapentadol—bulk substances	[50]
5 mM Britton Robinson buffer, pH 3.45, 14 kV, 16 °C, UV 214 nm	34 mM M-β-CD, 10 mM S-β-CD	sulpiride—bulk substance	[52]
25 mM phosphate, pH 7.0, 25 kV, 25 °C, UV 214 nm	10 mM β-CD, 15 mM SBE-β-CD	brompheniramine, chlorpheniramine, cetirizine, promethazine—bulk substances, tablets	[53]
100 mM phosphate, pH 3.5, 24 kV, 17 °C, UV 220 nm	10 mM β CD, 10 mM HP-β-CD (leucine) 10 mM β CD, 10 mM HP-γ-CD (phenylalanine)	benzothiazole derivatives of leucine and phenylalanine—bulk substances	[54]
50 mM phosphate, pH 3.0, 20 kV, 20 °C, UV 200 nm	5 mM S-β-CD, 0.1%b HS-γ-CD	ketamine metabolites—plasma samples	[56]
25 mM phosphate, pH 7.0, 20 kV, 18 °C, UV 210 nm	20 mM γ-CD, 10 mM SBE-β-CD (lansoprazole) 30 mM γ-CD, 15 mM SBE-β-CD (rabeprazole)	lansoprazole, rabeprazole—bulk substances	[57]
50 mM phosphate, pH 6.3, 20 kV, 20 °C, UV 210 nm	40.2 mg/mL DM-β-CD, 45 mM S-γ-CD	dapoxetine—chiral purity—bulk substance, tablets	[58]
30 mM phosphate, pH 6.5, 9 kV, 15 °C, UV 200 nm	14 mg/mL M-β-CD, 16 mg/mL S-β-CD	dextrometorphan—chiral purity—bulk substance, capsules	[59]
50 mM phosphate, pH 7.0, 25 kV, 15 °C, UV 200 nm	4 mM HP-β-CD, 16 mM TM-β-CD	phenoxy acid herbicides—bulk substances	[61]

## Abbreviations

The following abbreviations are used in this manuscript:

BGE	Background electrolyte
CD	Cyclodextrin
CE	Capillary electrophoresis
CM-β-CD	Carboxymethyl-β-CD
COX-2	Cyclooxygenase-2
CS	Chiral selector
DM-β-CD	Heptakis(2,6-di-O-methyl)-β-CD
DoE	Design of Experiments
HP-β-CD	Hydroxypropyl-β-CD
HP-γ-CD	Hydroxypropyl-γ-CD
HPLC	High performance liquid chromatography
HS-β-CD	Heptakis-6-sulfato-β-cyclodextrin
M-β-CD	Methyl-β-CD
MEKC	Micellar electrokinetic chromatography method
MPA-β-CD	6-monodeoxy-6-mono-(3-hydroxy)-propylamino-β CD
NH_2_-β-CD	Amino-β-cyclodextrin
NMR-	Nuclear magnetic resonance spectroscopy
NSAID	Non-steroidal anti-inflammatory drug
PIP-β-CD	Mono-6-deoxy-6-piperdine-β-cyclodextrin
PMMA-β-CD	Permethyl-6-monoamino-6-monodeoxy-β-CD
QbD	Quality by Design
S-β-CD	Sulfated-β-CD
SBE-β-CD	Sulfobutyl ether β-CD
TM-β-CD	Heptakis(2,3,6-tri-O-methyl)-β-CD
